# Raising Gene Therapy for Unmet Medical Needs in Japan

**DOI:** 10.31662/jmaj.2018-0040

**Published:** 2019-03-04

**Authors:** Tetsuya Ishii

**Affiliations:** 1 Office of Health and Safety, Hokkaido University, Sapporo, Japan

**Keywords:** gene therapy, ethics, regulation, cost, genetic disease, cancer, chimeric antigen receptor

## Abstract

Gene therapy has a complicated history. Some early trials resulted in catastrophes, including subjects’ deaths. In 2003, the world’s first gene therapy product (GTP) was approved in China. More recently, EU and US regulators have successively approved seven GTPs, including chimeric antigen receptor (CAR) T cells for refractory cancers and an adeno-associated vector, for treating serious genetic disease. In Japan, where there are no approved GTPs, some clinics have provided domestically-unapproved GTPs for cancer patients; however, in some cases, bereaved individuals litigated against such clinics. Meanwhile, the advent of GTPs is becoming controversial because of the unprecedentedly high treatment cost. The present article has three aims:

1) Reconsider the ethical legitimacy of gene therapy and its use for serious conditions.

2) Compare the Japanese, EU and US regulatory situations concerning GTPs and underscore Japan’s need for clearer and more up-to-date regulatory guidance.

3) Call for social understanding of GTPs and deliberations regarding the appropriate and acceptable cost, while noting that regulatory approval does not necessarily meet genetic disease patients’ needs.

To date, human genes are linked to at least 3,583 single gene diseases and traits ^(1)^. For such conditions, the transfer of a gene’s functional copy directly into a patient (*in vivo* gene therapy), and administration of genetically modified cells to a patient (*ex vivo* gene therapy), have been expected to be more effective than existing treatment modalities, such as chemical drugs. However, serious adverse events occurred in some early gene therapy trials, which were extremely concerning to regulators. In 2003, a Chinese regulator approved an adenovirus vector delivering *p53* for *in vivo* cancer gene therapy (*Gendicine*) as the world’s first gene therapy product (GTP); however, experts in other countries doubted its approval was justified because of the relatively small number of subjects ^(2)^. In 2012, the situation began to change. The European Medicines Agency (EMA) approved an adeno-associated virus (AAV) vector delivering *LPL* for treating lipoprotein lipase deficiency (LPLD)(*Alipogene tiparvovec, Glybera*); this was the first GTP approved in the West ^(3)^. Subsequently, the EU and US regulators have successively approved six GTPs for treating refractory cancers and genetic diseases. Of note, the US Food and Drug Administration (FDA) approved a robust cancer immunotherapy, Chimeric Antigen Receptor (CAR) T cells (*Tisagenlecleucel, KYMRIAH*) in 2017 ^(4)^, the EMA approved this *ex vivo* GTP in 2018 ^(5)^. Meanwhile, regulators and health care providers in Europe, the US, Japan, and other countries participate in the International Conference on Harmonization of Technical Requirements for Registration of Pharmaceuticals for Human Use (ICH) with the aim of harmonizing regulation of medical products, including GTPs (http://www.ich.org/products/consideration-documents.html). However, there are currently no approved GTPs in Japan. Meanwhile, *Glybera*, at a cost of 900,000 Euros (approximately 117,000,000 JPY) per patient, is currently the most expensive medicine ^(6)^. Worldwide, the extremely high treatment cost of *Glybera* and the expense of other GTPs has made them controversial. In 2018, an application for marketing *KYMRIAH* was made in Japan ^(7)^. It is now time to discuss how gene therapy would meet therapeutic needs in Japan. The present article first considers gene therapy’s legitimacy from an ethical standpoint. Then, it compares GTP’s regulatory situations in Japan to those in the EU and the US. Finally, it underscores the need for deliberations regarding the appropriate and acceptable cost of GTPs, in addition to social understanding of the limitations and risks in addition to benefits.

## Legitimacy of Gene Therapy

According to the Declaration of Helsinki, testing novel treatment modalities in humans is only permissible in cases in which the foreseeable benefits outweigh the predictable risks and burdens on the subjects. However, clinical uncertainty abounds if the risks are difficult to predict. This was the case with GTP development. Some adverse events occurred among the 2,597 gene therapy trials (http://www.abedia.com/wiley/indications.php). In 1999, a subject with ornithine transcarbamylase (OTC) deficiency died in a US *in vivo* gene therapy trial to assess the safety of an adenoviral vector carrying a functional copy of *OTC* ^(8)^. The tragedy occurred due to massive inflammatory responses that were elicited by the administered vector. Although the patient did not satisfy the inclusion criteria, the sponsor recruited him as a subject. Additionally, the risk of vector infusion observed in non-human primates was not disclosed to the patient. Thus, this case is characterized by the violation of research rules rather than safety issues inherent in gene therapy.

However, another catastrophic event was caused by gene transfer itself. In Europe, two *ex vivo* gene therapy trials were performed to treat life-threatening, X-linked, severe combined immunodeficiency (X-SCID) ^(9)^. Five of the 20 patients (4 in a Paris trial and 1 in a London trial) developed T-cell leukemia 2 to 5.5 years after the infusion of CD34+ cells retrovirally transduced with *IL2RG*. The retroviral vectors, which were inserted specifically near proto-oncogenes, caused the side effect of leukemia. After chemotherapy, four patients survived and showed sustained remission and immunity; however, one patient died from refractory leukemia. In a US trial of an AAV vector to treat hemophilia B, viral DNA contamination was found in the semen of a male subject in whom the vector had been administered hepatically ^(10)^; this is the so-called viral vector shedding issue, which can lead to inadvertent integration of viral DNA in the germline. More significantly, in 2007, a subject died in a US trial using an AAV vector to treat rheumatoid arthritis (RA) ^(11)^. Since the vector was designed to express an immunoglobulin domain fused to a TNF-receptor domain, its causal role was unclear but could not be ruled out. Moreover, preclinical animal experiments did not predict such risks at all.

However, there are currently countermeasures against these risks. Lessons from the French X-SCID trial led to an improved retrovirus vector containing deletions in viral enhancer sequences, which was safely used in a recent similar multi-center trial ^(12)^. Regarding the vector shedding issue, monitoring vector excretion from the body is now required; enrolled patients should take contraceptive measures during trials ^(13)^. The adverse event following the RA trial suggests there is still some uncertainty in gene therapy trials, even when the trial uses AAV vectors that are believed to be almost nonpathogenic to humans. On closer examination, RA patients are also medically unstable. Given that either viral vector administration or irreversible genetic intervention both involve substantial risks, RA -a non-lethal condition- is not considered a compelling condition for testing gene therapy since the risks outweigh the potential benefits. Conversely, if a careful prior risk assessment and a meticulous follow-up of subjects are made, gene therapy testing would be permissible for more serious conditions. In addition, only genetic interventions can provide fundamental treatments for a genetic disease caused by a gene mutation. Thus, clinical development of gene therapy is generally considered legitimate for serious conditions, particularly life-threatening genetic diseases that have no fundamental treatments, under an appropriate risk to benefit ratio.

## The Regulatory Situations in Japan, Europe, and the US

**Figure 1.**
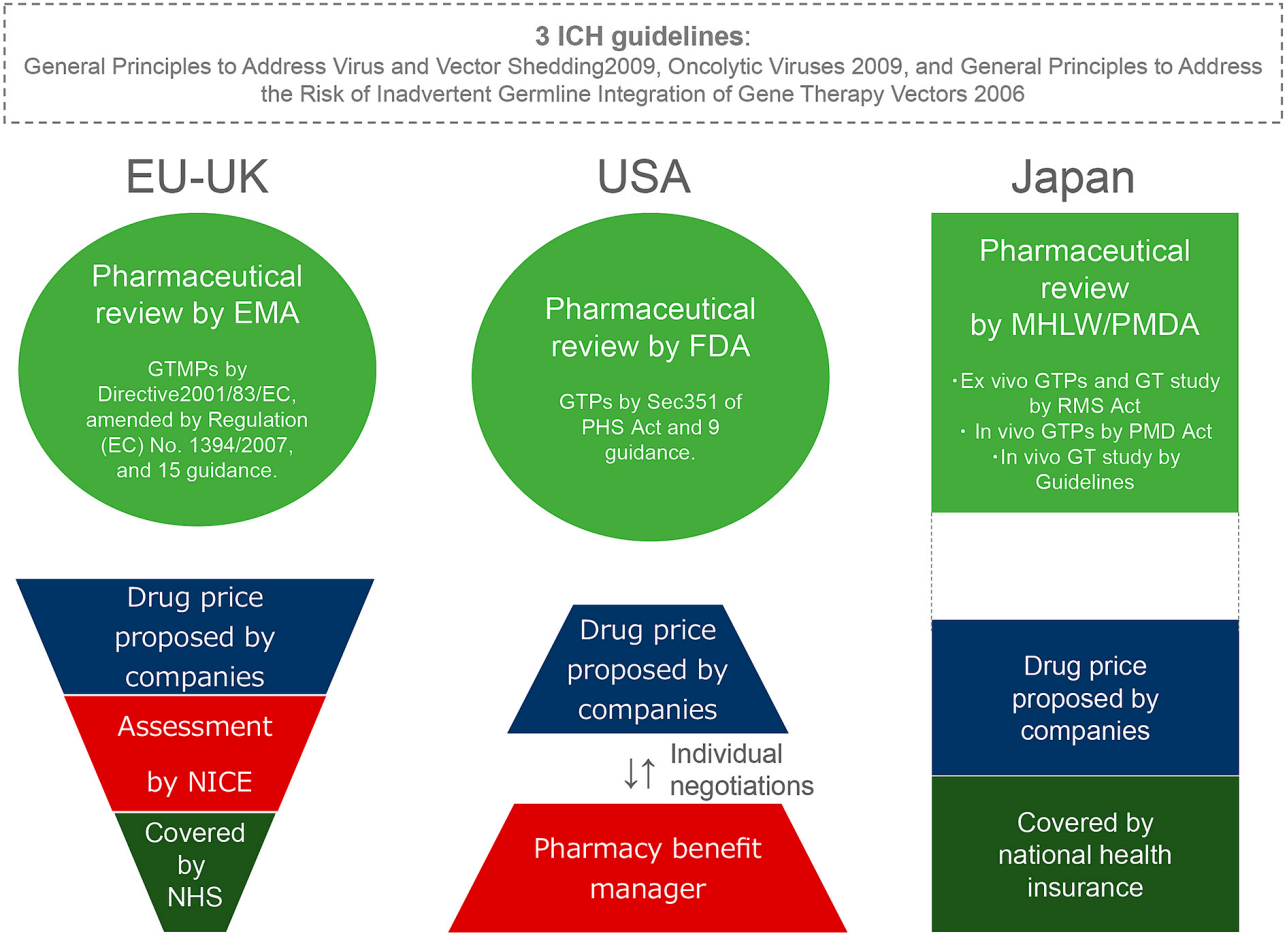
The regulatory approaches to, and pricing of, gene therapy products (GTPs) in the EU, the US and Japan. The European Medicines Agency (EMA) regulates Gene Therapy Medicinal Products (GTMPs) as one of the Advanced Therapeutic Medicinal Products (ATMPs), including Somatic Cell Therapy Medicinal Products, Tissue Engineered Products, and Combined ATMPs (medical products combining one or more of the previous categories), under Directive 2001/83/EC, as amended by Regulation (EC) No. 1394/2007, with 15 guidance other than the three ICH guidelines. The US Food and Drug Administration (FDA) largely categorizes biologics into lower risk and higher risk products under the Public Health Services (PMS) Act. Lower risk products include human cells, tissues, and cellular and tissue-based products (HCT/Ps) that meet the criteria stipulated by Section 361 of the PMS Act: minimal manipulation; homologous use; not combined with another article (product); either no systemic effect and do not rely on metabolic effect of living cells; or have a systemic effect and rely on metabolic effect of living cells but are for autologous, first or second-degree blood relative or reproductive use. GTPs are classified as higher risk biologics that do not meet the criteria stipulated by Section 361. The EU and US regulate *in vivo* and* ex vivo* GTPs within the general regulatory framework, issuing instructive guidance, whereas Japan regulates gene therapy using three different regulatory tracks. If a GTP receives marketing approval from the EMA, the pharmaceutical company can negotiate the price with each EU member state. In the UK, the National Institute for Health and Care Excellence (NICE) assesses the price of a proposed drug based on the principle of cost-effectiveness. National Health Service coverage is only extended to drugs when NICE’s assessment of the price is favorable. In the US, pharmaceutical companies individually negotiate drug prices with pharmacy benefit managers based on economic liberalism. In Japan, the price of a drug that is to be covered by national health insurance is generally proposed by pharmaceutical companies. However, the cost-effectiveness will likely be considered in pharmaceutical pricing.

As mentioned above, Japanese Ministry of Health, Labour, and Welfare (MHLW), the EMA and the US FDA participate in the ICH and share three gene therapy guidelines regarding viral vector shedding and the risks of inadvertent germline integration and oncolytic viruses (Figure 1, http://www.ich.org/products/consideration-documents.html). However, the situations of GTP approval differ between Japan and the two other jurisdictions. The European and US regulators have approved seven GTPs (*ex vivo*: 4, *in vivo*: 3) to treat cancers and genetic diseases (Table 1). Notably, one GTP for treating retinal dystrophy: *Voretigene neparvovec-rzyl (LUXTURNA)*, as well as three cancer GTPs: *talimogene laherparepvec* (*IMLYGIC*), *KYMRIAH*, and *Axicabtagene ciloleucel (YESKARTA)*, are approved in both the US and the EU. However, at the time of this writing, there are no approved GTPs in Japan.

**Table 1.** Gene Therapy Products Approved in the EU and the USA (as of January 24, 2019).

Descriptive name (Trade name)	Jurisdiction approved in	Developmental incentives	Intervention type	Indications	Remarks
Alipogene tiparvovec (Glybera)	EU 2012	Orphan Medicine, Conditional Marketing Approval.	*In vivo* gene therapy	Severe pancreatitis due to lipoprotein lipase deficiency (LPLD)	A non-integrative AAV1 vector is administered intramuscularly to deliver a normal copy of LPL gene to muscle cells. However, its marketing was withdrawn in 2017.
Talimogene laherparepvec (IMLYGIC)	USA 2015, EU in 2015	Special Protocol Assessment, Priority Review, Orphan Drug (USA)	*In vivo* gene therapy	Melanoma	An oncolytic HSV1 is directly injected into patients’ melanoma tumors, delivering GM-CSF gene to the cells.
Autologous CD34+ enriched cell fraction that contains CD34+ cells transduced with retroviral vector that encodes for the human ADA cDNA sequence (Strimvelis)	EU 2016	Orphan Medicine, Paediatric Investigation Plan	*Ex vivo* gene therapy	Severe combined immunodeficiency due to adenosine deaminase deficiency (ADA-SCID), where bone-marrow transplants are unavailable.	A retrovirus vector delivers a normal copy of ADA gene to CD34+ cells. The modified cells are infused intravenously.
Allogeneic T cells genetically modified with a retroviral vector encoding for a truncated form of the human low affinity nerve growth factor receptor (ΔLNGFR) and the herpes simplex I virus thymidine kinase (HSV-TK Mut2) (Zalmoxis)	EU 2016	Orphan Medicine	*Ex vivo* gene therapy	Prevention of complications in hematopoietic stem cell Transplantation.	Allogeneic T cells are genetically modified with a retroviral vector coding HSV-TK and ΔLNGFR genes so that these genes are integrated in the genome of the cells. If graft vs host disease (GvHD) occurs, the cell product can be inactivated by administrating ganciclovir.
Tisagenlecleucel (KYMRIAH)	USA 2017, EU 2018	Priority Review, Breakthrough Therapy, Orphan Drug (USA)	*Ex vivo* gene therapy	Patients up to 25 years of age with B-cell precursor acute lymphoblastic leukemia (ALL) that is refractory or in second or later relapse.	Autologous T cells are genetically modified using a lentiviral vector to encode an anti-CD19 CAR, including a murine anti-CD19 single chain antibody fragment (scFv) and two human signaling domains (CD3-ζ and 4-1BB). The modified cells are administered intravenously.
				Adult patients with relapsed or refractory large B-cell lymphoma after two or more lines of systemic therapy.	
Axicabtagene ciloleucel (YESKARTA)	USA 2017, EU 2018	Priority Review, Breakthrough Therapy, Orphan Drug (USA)	*Ex vivo* gene therapy	Adult patients with relapsed or refractory large B-cell lymphoma after two or more lines of systemic therapy.	Autologous T cells are genetically modified, using a lentiviral vector, to encode an anti-CD19 CAR, including a murine scFv and two human signaling domains (CD3-ζ and CD28). The CAR T cells are administered intravenously.
Voretigene neparvovec-rzyl (LUXTURNA)	USA 2017, EU 2018	Priority Review, Breakthrough Therapy, Orphan Drug, Rare Pediatric Disease Designation (USA)	*In vivo* gene therapy	Patients with biallelic RPE65 mutation-associated retinal dystrophy.	A non-integrative AAV2 vector is administered subretinally to deliver a normal copy of RPE65 gene to retinal cells.

AAV: adeno-associated virus, CAR: chimeric antigen receptor, HSV: Herpes Simplex Virus, GM-CSF: granulocyte macrophage colony-stimulating factor

To gain regulatory insight, the regulatory approaches of three jurisdictions to GTPs are discussed below. The EU and the USA have adopted a similar general regulatory framework to regulate GTPs as a relatively new type of biologics ^(14)^. The EMA regulates GTPs as Gene Therapy Medicinal Products (GTMPs), which are Advanced Therapeutic Medicinal Products (ATMPs) under *Directive 2001/83/EC*, amended by *Regulation (EC) No. 1394/2007*. For proper GTMP regulation, the EMA has issued 15 guidance statements (other than the three ICH guidelines) since 2001 ^(14)^. Moreover, development of *Glybera*, an *ex vivo* GTP for treating severe combined immunodeficiency due to adenosine deaminase deficiency (*Strimvelis*) and an *ex vivo* GTP for preventing complications in hematopoietic stem cell transplantation (*Zalmoxis*) were encouraged by designating these GTPs as *Orphan Medicine* and/or *Pediatric Investigation Plan* (Table 1).

In the US, biologics are largely categorized into lower risk and higher risk medical products under the *Public Health Services (PMS) Act*. Section 351 of the *PMS Act* classifies GTPs as higher risk biologics that do not meet Section 361 criteria because genetic interventions are not considered minimal manipulation and involve either systemic or metabolic effects on living cells. As gene therapy technically evolved, the FDA issued nine guidance statements regarding GTPs ^(14)^. In addition, the FDA promoted developing *IMLYGIC, KYMRIAH, Axicabtagene ciloleucel* (*YESKARTA*: a CAR T-cell product) and *Voretigene neparvovec-rzyla* (*LUXTURNA*: an *in vivo* GTP for treating a mutation-associated retinal dystrophy) by designating those GTPs as *Breakthrough Therapy, Orphan Drug, Priority Review, Rare Pediatric Disease,* and/or *Special Protocol Assessment* (Table 1). Thus, the EU and USA regulate *in vivo* and *ex vivo* GTPs within their general regulatory frameworks as other types of biologics (Figure 1), issuing instructive guidance and various incentives for developers.

In contrast, Japan has regulated gene therapy in a different manner from the EU and the US. Firstly, clinical research is classified differently from clinical trials in the Japanese regulatory system; clinical trials aim to obtain regulatory approvals for manufacturing and marketing medical products, whereas clinical research aims to gain medical data. With regard to gene therapy, the MHLW and an affiliate regulator, the Pharmaceuticals and Medical Devices Agency, regulate gene therapy using three tracks:

1) *Regenerative Medicine Promotion (RMP) Law 2013* and *the Act of Safety of Regenerative Medicine 2013* (RMS Act) to regulate regenerative medicine products and relevant clinical research, including *ex vivo *GTPs;

2) *The Act on Pharmaceuticals and Medical Devices* (PMD Act) 2013 to regulate a wide range of medical products, including *in vivo* GTPs; and

3) *The Guidelines of Clinical Research Regarding Gene Therapy 2015* for regulating clinical research on *in vivo* gene therapy (Figure 1). The clinical research on *ex vivo* and *in vivo* gene therapies aims to gain medical data on gene therapy. As such, the sponsors are largely university physicians, not in the private sector.

To seek manufacturing and marketing authorization, *ex vivo* GTPs shall be developed in compliance with *RMP* Law and the *RMS* Act, whereas *in vivo* GTPs shall be developed in accordance with the *PMD* Act. The *RMS* Act categorizes three Regenerative Medicine Products (RMPs) based on potential risks, similar to the US regulatory framework. Among the three, *ex vivo* GTPs, as well as induced pluripotent stem cells, are classified as the highest risk RMPs. In Japan, where *Priority Review Designation* and *Rare Disease Designation* are already available, *Conditional Prompt Marketing Approval Designation* was introduced in 2017. This allows developers to apply conditional marketing approval without performing costly phase III trials if enough patients with a rare disorder cannot be recruited ^(15)^. Meanwhile, since GTPs are a relatively new category of biologics, their development requires updated and clear guidance based on regulatory science. However, Japan currently has little guidance clarifying GTP regulation except for three relevant ICH guidelines. The MHLW had one GTP-specific guidance statement (Notification No.1062 of 1995: the *Guidance for Ensuring the Quality and Safety of Medical Products for Gene Therapy*); however, the health ministry repealed it to shift from document-based guidance to consultation-based guidance ^(16)^. The fact that there are no approved GTPs in Japan suggests that consultation-based guidance is unclear and not so instructive for developers. Moreover, newer gene-editing techniques are being integrated into gene therapy ^(17)^, demanding regulatory guidance on the existing three gene therapy tracks. Thus, the Japanese regulatory approach to GTPs is more complicated and less instructive for developers than the European and US regulatory frameworks.

Some experts in Japan ^(18)^ pointed out that another regulatory issue, the Act on the Conservation and Sustainable Use of Biological Diversity through Regulations on the Use of Living Modified Organisms 2003 (so-called Cartagena Law), imposed huge vector management burdens on gene therapy developers in clinical settings. Moreover, they pointed out lower public funding for gene therapy, fewer researchers, and an insufficient system for manufacturing clinical-grade viral vectors. These issues have delayed GTP development in Japan.

## Cost and Access

Developing advanced medicine requires tremendous research investments. If a treatment modality targets a rare disease, the smaller patient population generally makes it difficult to recover the initial research investment. Moreover, manufacturing biologics, including GTPs, is largely less efficient than chemical agents. In addition, the longer expected efficacy and the fact that GTPs are administered fewer times make the cost of such treatments more expensive, particularly for rare diseases.

*Glybera* was administered to one LPLD patient in Germany, and greatly alleviated his condition ^(19)^. However, the GTP has only been used once since its approval in 2012 because it is so expensive. Moreover, *Glybera* failed to enter the US market due to FDA skepticism of European clinical data ^(6)^. Eventually, the provider abandoned the project and discontinued marketing authorization in 2017 ^(3)^, so current and future LPLD patients will no longer benefit from *Glybera*. In 2018, another enterprise announced the transfer of its program of investigational and approved rare disease GTPs, including *Strimvelis,* to a corporation ^(20)^. Although the UK National Institute for Health and Care Excellence (NICE) recommended a treatment cost of €594,000 (JPY 77,220,000) per patient ^(21)^, the enterprise eventually decided to shift its focus from rare diseases. Such complications in the EU, in which member states largely provide national health insurance rather than the market-driven health insurance system adopted in the USA, suggest that the marketing approval of GTPs for rare genetic diseases does not necessarily meet therapeutic needs. This is also the case in Japan, which also provides national health insurance (Figure 1).

In contrast, an* ex vivo* cancer GTP, *KYMRIAH,* expanded its market from the US to the EU. The population of *KYMRIAH* patients is larger than the populations targeted by GTPs for rare genetic disease, such as *Glybera* and *Strimvelis,* because it has two approved cancer indications (Table 1). In 2018, a marketing application for *KYMRIAH* was also made in Japan. If approved, the cost proposed to the MHLW may be equivalent to, or less than, the cost (￡282,000: approximately JPY 41,740,000 per patient) that UK NICE once rejected due to low cost-effectiveness ^(22)^. Notably, the treatment cost (JPY 35,000,000/patient/year) of a cancer antibody medicine, *Nivolumab* (*Opdivo*), was hotly debated in the MHLW, which led to a great reduction of the price ^(23)^. However, reducing the price of a new medicine too much will discourage pharmaceutical companies from developing advanced medicines, and likely hamper the advent of GTPs, particularly for rare genetic diseases. Meanwhile, some clinics have provided domestically-unapproved cancer GTPs as free medical care in Japan. However, some bereaved families sued one of the clinics for damages, claiming that they had paid as much as JPY 5,400,000 for the unproven GTP ^(24)^. This problem could reflect either the patient’s overestimation of the efficacy and/or inappropriate informed consent regarding GTPs. Taken together, these unsolved issues suggest that Japan currently has difficulties with GTP pricing and approval.

## Conclusion

This article asserts that gene therapy, which transfers a functional copy of a gene to humans, is ethically legitimate in serious conditions, particularly for life-threating genetic diseases that develop due to genetic mutations. Clear and updated regulatory guidance is needed to accelerate GTP development in Japan. Furthermore, the regulatory approval of GTPs does not necessarily meet a society’s therapeutic needs. There is a pressing need for deliberations regarding the GTPs’ appropriate and acceptable costs that can be harmonized with the national health insurance system. In the process, special care should be taken, as the social integration of GTPs for treating rare genetic diseases is more challenging than those treating refractory cancers. In addition, the public should be carefully informed that most GTPs involve limitations and risks, in addition to their more prolonged efficacy.

## 

Note. On Feb 20, 2019, a technical committee of MHLW recommended the approval of two GTPs: *KYMRIAH* and an *in vivo* GTP for HGF gene transfer in patients with a peripheral arterial disease.

The indications of two GTPs are serious conditions but not genetic disease.

## Article Information

### Conflicts of Interest

None

### Sources of Funding

This work was supported by the funding from Hokkaido University.

### Author Contributions

TI conceived the concept, prepared the figure and table, and wrote the manuscript.
